# Occult *Talaromyces marneffei* Infection Unveiled by the Novel Mp1p Antigen Detection Assay

**DOI:** 10.1093/ofid/ofaa502

**Published:** 2020-10-19

**Authors:** Vo Trieu Ly, Nguyen Tat Thanh, Nguyen Thi Mai Thu, Jasper Chan, Jeremy N Day, John Perfect, Cao Ngoc Nga, Nguyen Van Vinh Chau, Thuy Le

**Affiliations:** 1 University of Medicine and Pharmacy at Ho Chi Minh City, Ho Chi Minh City, Vietnam; 2 Hospital for Tropical Diseases, Ho Chi Minh City, Vietnam; 3 Oxford University Clinical Research Unit, Ho Chi Minh City, Vietnam; 4 State Key Laboratory of Emerging Infectious Diseases, Carol Yu Centre for Infection, Department of Microbiology, Li Ka Shing Faculty of Medicine, The University of Hong Kong, Pokfulam, Hong Kong; 5 Hainan Medical University–The University of Hong Kong Joint Laboratory of Tropical Infectious Diseases, Hainan Medical University, Haikou, Hainan, China; 6 Center for Tropical Medicine and Global Health, Nuffield Department of Medicine, University of Oxford, Oxford, UK; 7 Division of Infectious Diseases and International Health, Duke University School of Medicine, Durham, North Carolina, USA

**Keywords:** Mp1p EIA, *Penicillium marneffei*, penicilliosis, *Talaromyces marneffei*, talaromycosis

## Abstract

*Talaromyces marneffei* causes fatal invasive mycosis in Southeast Asia. Diagnosis by culture has limited sensitivity and can result in treatment delay. We describe the use of a novel Mp1p enzyme immunoassay (EIA) to identify blood culture–negative talaromycosis, subsequently confirmed by bone marrow cultures. This EIA has the potential to speed diagnosis, enabling early therapy initiation.

Talaromycosis (penicilliosis) is an invasive mycosis caused by the dimorphic fungus *Talaromyces marneffei* (Tm), which is highly endemic in Southeast Asia [1], and has been diagnosed worldwide in returning travelers and immigrants from the region [2, 3]. Talaromycosis primarily affects individuals with advanced HIV disease, in whom it ranks as the third most common opportunistic infection, and is a leading cause of HIV-associated death [4–6]. Incidence is rapidly increasing in non-HIV-infected individuals who have malignancies, receive immunosuppressive therapy, or receive solid organ or bone marrow transplantations [7]. Human infection results from inhalation of aerosolized fungal spores from the environment. Infection can remain latent for years and can be reactivated in immunosuppressed individuals, causing disseminated disease involving the lungs, gastrointestinal tract, lymphatics, liver, spleen, blood, skin, and bone marrow [8]. The mortality rate despite antifungal therapy is up to 30% in HIV-infected [4, 5, 6] and 50% in non-HIV-infected individuals [9].

The gold standard for diagnosis is culture. However, this is poorly sensitive and takes up to 2 weeks to yield a positive result, leading to treatment delay [1, 8]. Identifying early disease, where interventions are most likely to be effective, is particularly challenging. While diagnosis can be made based on microscopy of lymph nodes or skin lesions, such diagnostic lesions are present in only 30% to 50% of patients [5, 9]. Blood culture remains the mainstay of diagnosis but misses 30% of infections in HIV-infected [5, 10] and 50% in non-HIV-infected patients [9]. A clinical trial cohort from Vietnam identified diagnostic delays of up to 6 months among patients [11]. Another cohort from China found diagnostic delays to be associated with increases in mortality from 24.3% to 50.6% [4]. Our research group has developed a novel monoclonal antibody–based enzyme immunoassay (EIA) that detects a Tm-specific protein Mp1p located throughout the cell wall of Tm [12, 13]. This antigen is abundantly secreted in the blood and urine of patients during infection, and we have recently demonstrated that this Mp1p EIA is more sensitive than conventional BACTEC blood cultures in detecting talaromycosis [14]. Here, we report 2 HIV-infected patients who presented with recurrent fevers and sepsis of unclear etiology, in whom multiple cultures of blood and other specimens were negative for pathogens. The diagnosis of talaromycosis was made based on a positive Mp1p EIA, which was confirmed by microscopy and cultures of bone marrow. The cases illustrate important features of this neglected infectious disease and the role of antigen detection in making early diagnosis.

## PATIENT 1

Patient 1 was a 35-year-old man, an intravenous drug user, who presented to the Hospital for Tropical Diseases in Ho Chi Minh City (HCMC) with a 1-week history of high fevers, fatigue, vomitting, and diarrhea. He had been hospitalized 2 weeks before for fevers and was treated empirically for bacterial sepsis. He had been diagnosed with HIV infection 10 years previously, received antiretroviral therapy (ART) with tenofovir, lamivudine, and efavirenz, and had been switched to second-line therapy with tenofovir, lamivudine, and lopinavir/ritonavir 1 month before this admission due to treatment failure. His CD4+ T-cell count at that time was 10 cells/µL. He had recently completed therapy for tuberculous adenitis. On admission, he had pyrexia of 39°C, confusion, several shallow nontender ulcers on the hard palace, an enlarged cervical lymph node of 2 cm, and mild hepatosplenomegaly. His skin was normal. Laboratory tests revealed pancytopenia (white cells 2.6 × 10^9^ L, hemoglobin 11 g/dL, platelets 43 × 10^9^ L) and mild transaminitis (aspartate transaminase 64 U/L, alanine transaminase 54 U/L). Chest x-ray was normal. Abdominal ultrasound confirmed hepatosplenomegaly. He underwent a lumbar puncture. Cerebrospinal fluid (CSF) cell counts and chemistry were normal. CSF Indian ink and CSF culture were negative. Multiple sputum smears and cervical lymph node aspirate were negative for acid-fast bacilli (AFB). No pathogens were identified from 3 sets of blood cultures using the automated BACTEC system after 5 days of culture and stool cultures. He was again treated for suspected bacterial sepsis with 8 days of imipenem but remained febrile with no clinical improvement.

## PATIENT 2

Patient 2 was a 28-year-old HIV-infected man referred to our hospital with 3 months of worsening fevers, fatigue, exertional dyspnea, and weight loss. His symptoms began within 1 week of initiating ART with tenofovir, lamivudine, and efavirenz. His CD4+ T-cell count at that time was 19 cells/µL. Outpatient work-up included multiple negative sputum AFB smears, negative blood cultures, and normal chest x-rays. On admission he had pyrexia of 38.0°C and cachexia. There was no lymphadenopathy; his skin was normal. He had severe pancytopenia (white cells 0.9 × 10^9^ L, hemoglobin 8.3 g/dL, platelets 21 × 10^9^ L). Liver enzymes were normal. Abdominal ultrasound revealed hepatosplenomegaly and multiple intra-abdominal lymph nodes that were 1–2 cm in diameter. Multiple sputum AFB smears and blood cultures were again negative. Chest x-ray was normal. The patient was treated for suspected bacterial sepsis with ceftriaxone for 7 days without improvement.

## STATEMENT OF PATIENT CONSENT AND ETHICS

Both patients gave written consent and participated in a prospective study using the novel Mp1p EIA to screen for talaromycosis in hospitalized patients with AIDS. They gave a separate written consent for this report. The study was approved by the Hospital for Tropical Diseases (approval number 52/HDDD; November 30, 2018).

**Figure 1.**
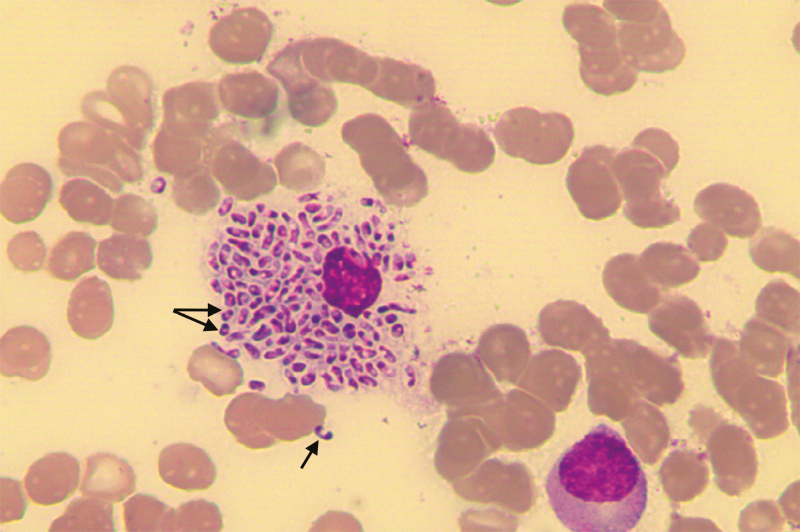
Wright’s stained smear of the bone marrow aspirate of Patient 1 showing numerous yeast cells measuring 5–6 µm inside an engorged histiocyte. The arrows show the actively dividing yeast cells, revealing a midline septum characteristic of *Talaromyces marneffei*.

**Figure 2.**
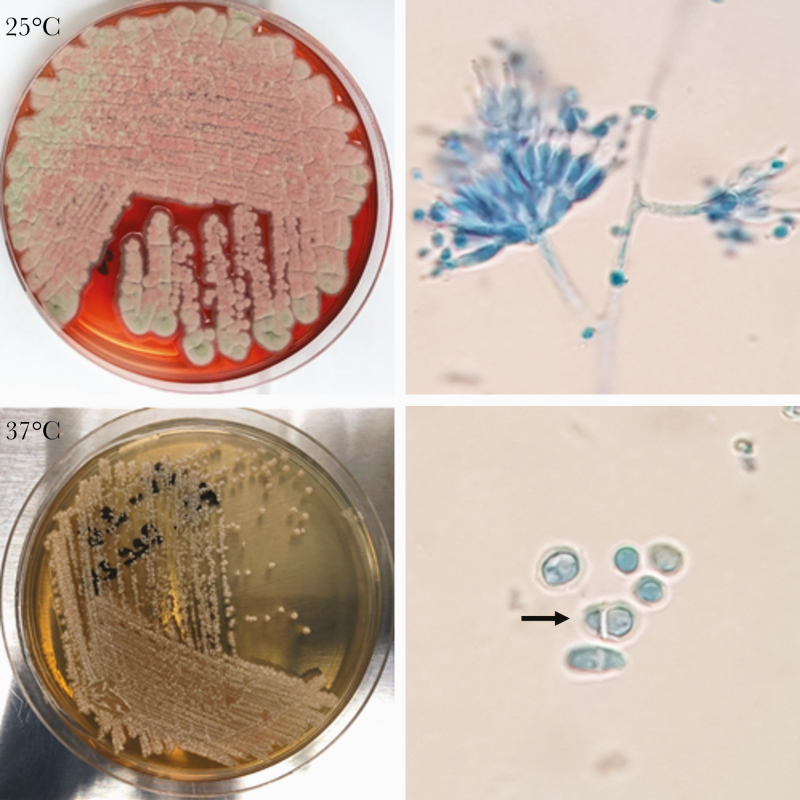
*Talaromyces marneffei* subcultures from the bone marrow of Patient 2. At 25°C, *T. marneffei* produces powdery greenish mold colonies and a bright red pigment that diffuses into Sabouraud Dextrose Agar (SDA) medium. Tape preparation of the mold colonies shows septate hyphae with conidiophores bearing phialides and round conidia under the microscope. At 37°C, *T. marneffei* produces white yeast colonies on SDA media without the red pigmentation. Microscopic examination shows oval yeast cells, 1 with a central septum (arrow).

Based on an established optical density (OD) cutoff of 0.5^15^, the EIA tests were positive in the serum, plasma, and urine samples of both patients (Patient 1: ODs = 1.05, 1.91, 2.84; Patient 2: ODs = 2.96, 3.07, 2.92, respectively). As the Mp1p EIA was being evaluated in a research study, antifungal therapy was not yet started. However, the study protocol dictated that a bone marrow biopsy be offered to evaluate for deep-seated infection in all antigen-positive culture-negative patients who also had evidence of bone marrow suppression. The microscopy of the bone marrow aspirates in both patients showed numerous intra- and extracellular yeast cells, some revealing a mid-line septum characteristic of Tm (Figure 1). Treatment with amphotericin B deoxycholate was initiated. Both patients had rapid improvement after 1 week of therapy. Amphotericin B was switched to itraconazole early in patient 1 due to reduced creatinine clearance, but he continued to improve and was discharged home. Patient 2 developed a suswedden onset of severe abdominal pain and distention. Abdominal x-ray showed free air. He unfortunately succumbed to septic shock due to suspected peforated viscous. Tm was isolated from bone marrow cultures of both patients (Figure 2).

## DISCUSSION

These cases demonstrate several important features of talaromycosis. First, infection has an indolent course with nonspecific signs and symptoms that are indistinguishable from infections due to salmonellosis, invasive mycoses, tuberculosis, and other mycobacteria. Further, coinfections with these pathogens occur frequently in this profoundly immunosuppressed population [5, 10], posing a challenge for diagnosis and management. Second, even in the settings of advanced HIV disease and disseminated infection, blood cultures can be negative in talaromycosis. While a bone marrow biopsy is clinically indicated, these are rarely performed in low-income, high–tuberculosis burden settings such as ours. Was it not for the Mp1p EIA performed in these cases, the diagnosis would have been missed; the most likely outcome would have been commencement of 9 months of antituberculosis chemotherapy. This would almost certainly have resulted in the death of Patient 1. These cases highlight the need for rapid, more sensitive, non-culture-based diagnostics for talaromycosis. Several real-time polymerase chain reaction (PCR) assays have been developed to detect Tm directly from blood [15–18]; however, their sensitivities have been suboptimal (60%–85%) due to DNA loss during extraction. EIA-based antigen detection has become a standard diagnostic for other mycoses including aspergillosis, cryptococcosis, and histoplasmosis [19–21]. Among 2 recently developed monoclonal antibody–based assays that detect Tm antigen [14, 22], our Mp1p EIA is advantageous, as it targets a Tm-specific antigen abundantly secreted in the blood and urine of patients. In our study of 372 culture-positive cases and 517 controls, we have demonstrated that the Mp1p EIA is more sensitive than blood cultures (86.3% vs 72.8%; *P* < .001) and has a specificity of 98.1% [14]. The test is more sensitive in urine than in plasma specimens, and testing plasma and urine in combination further increases sensitivity [14]. Our patients had high Mp1p ODs in the urine, higher than in the serum and plasma of Patient 1. This supports our previous finding and suggests that, similar to histoplasmosis, urine is an excellent specimen for Tm antigen testing. Our cases illustrate how the Mp1p EIA can guide management of immunocompromised patients living in or having traveled to Tm-endemic countries who present with undifferentiated fevers. Our cases provide further support for the Mp1p EIA as a rapid diagnostic tool for talaromycosis. A prospective Tm antigen screening study is underway to define the diagnostic utility of antigen testing in populations at risk for talaromycosis (ClinicalTrials.gov: NCT04033120).

Finally, these cases demonstrate how ART unmasks talaromycosis in patients with advanced HIV disease. Both patients developed symptoms of infection within 1 week of initiating ART. Such unmasking immune reconstitution inflammatory syndrome (IRIS) is frequently seen in talaromycosis [23–25]. Among 440 talaromycosis patients who participated in our itraconazole vs amphotericin B clinical trial (IVAP) [11], 40% developed talaromycosis within 4 weeks of ART initiation. We believe there is a role for antigen screening in detecting subclinical infections at the time of ART initiation. This may permit prevention of disease through preemptive antifungal therapy. Such a screen-and-preemptive strategy has been shown to reduce mortality and be cost-effective in HIV-associated cryptococcal meningitis [21, 26–28] and can serve as a model for talaromycosis prevention.
